# Partial Substitution of K by Na Alleviates Drought Stress and Increases Water Use Efficiency in *Eucalyptus* Species Seedlings

**DOI:** 10.3389/fpls.2021.632342

**Published:** 2021-03-15

**Authors:** Nikolas de Souza Mateus, Antônio Leite Florentino, Elcio Ferreira Santos, Alexandre de Vicente Ferraz, José Leonardo de Moraes Goncalves, José Lavres

**Affiliations:** ^1^Stable Isotope Laboratory, Center for Nuclear Energy in Agriculture, University of São Paulo, Piracicaba, Brazil; ^2^Applied Ecology Laboratory, Department of Forest Sciences, Luiz de Queiroz College of Agriculture, University of São Paulo, Piracicaba, Brazil; ^3^Federal Institute of Mato Grosso Do Sul, Nova Andradina, Brazil; ^4^Institute of Forest Research and Studies, Piracicaba, Brazil

**Keywords:** sodium application, drought, stable carbon isotope, water use efficiency, water consumption, photosynthesis

## Abstract

*Eucalyptus*, the most widely planted tree genus worldwide, is frequently cultivated in soils with low water and nutrient availability. Sodium (Na) can substitute some physiological functions of potassium (K), directly influencing plants’ water status. However, the extent to which K can be replaced by Na in drought conditions remains poorly understood. A greenhouse experiment was conducted with three *Eucalyptus* genotypes under two water conditions (well-watered and water-stressed) and five combination rates of K and Na, representing substitutions of 0/100, 25/75, 50/50, 75/25, and 100/0 (percentage of Na/percentage of K), to investigate growth and photosynthesis-related parameters. This study focused on the positive effects of Na supply since, depending on the levels applied, the Na supply may induce plants to salinity stress (>100 mM of NaCl). Plants supplied with low to intermediate K replacement by Na reduced the critical level of K without showing symptoms of K deficiency and provided higher total dry matter (TDM) than those *Eucalyptus* seedlings supplied only with K in both water conditions. Those plants supplied with low to intermediate K replacement by Na had improved CO_2_ assimilation (*A*), stomatal density (Std), K use efficiency (UE_*K*_), and water use efficiency (WUE), in addition to reduced leaf water potential (*Ψ*w) and maintenance of leaf turgidity, with the stomata partially closed, indicated by the higher values of leaf carbon isotope composition (δ^13^C‰). Meanwhile, combination rates higher than 50% of K replacement by Na led to K-deficient plants, characterized by the lower values of TDM, δ^13^C‰, WUE, and leaf K concentration and higher leaf Na concentration. There was positive evidence of partial replacement of K by Na in *Eucalyptus* seedlings; meanwhile, the ideal percentage of substitution increased according to the drought tolerance of the species (*Eucalyptus saligna* < *Eucalyptus urophylla* < *Eucalyptus camaldulensis*).

## Introduction

The genus *Eucalyptus* plays an important role in meeting the growing global wood demand (Paquette and Messier, 2010). However, it is largely dependent on fertilization (Smethurst, 2010) and vulnerable to drought, the main limiting factors for plant growth (Gonçalves et al., 2017). Against the background of a changing climate, the intensity, and frequency of drought will increase in the near future (IPCC, 2019). Adequate management strategies to improve tolerance to water deficit, such as enhancing plant water use efficiency (WUE), are necessary to mitigate the adverse impacts of drought (Asensio et al., 2020). Stomatal closure by osmotic adjustment (Oddo et al., 2011) is a key factor to mitigating the negative impacts of drought, avoiding excessive water loss at the expense of photosynthetic rate restrictions (Anjum et al., 2011) and turgor loss, decreasing cell growth (Steudle, 2000). Among the macronutrients, potassium (K) is one of the most required nutrients for *Eucalyptus*, enhancing yields by 50% compared to plants under K deficiency (Battie-Laclau et al., 2013). Changes in cell turgor involve the controlled uptake of K and other ions, mediated by voltage-gated K^+^ transporters at the cellular plasma membrane, inducing solute accumulation (Shabala and Lew, 2002), water uptake from the apoplast, and, finally, stomata aperture (Ahmad and Maathuis, 2014). Thus, the ion flux in and out of the guard cells mediates stomatal aperture and closure (Kim et al., 2010).

Sodium (Na), a beneficial element, is absorbed and taken up as Na^+^ and might replace K partially as an osmotically active solute, stimulating cell elongation and improving stomatal control, which, in turn, contributes to maintain cell turgor (Jeschke, 1977), directly affecting plant WUE (Mateus et al., 2019). Additionally, some ATPases require both K and Na for maximal activity (Marschner, 2012). This occurs due to the similarity between the hydrated ionic radii of Na (0.358 nm) and K (0.331 nm) (Marschner, 2012). Despite the well-known importance of K, the effects of Na application on water balance are not well studied (Gattward et al., 2012). A major benefit of replacing K fertilization by Na is the relatively lower cost of NaCl compared to KCl, bringing greater profitability to the forest sector; besides, nutrient interaction may be a strategy to increase K use efficiency and decrease the critical K leaf concentration (Laclau et al., 2003). K deficiency reduces plant tolerance to water deficit due to its influence on plant osmoregulation, playing a critical role in stress avoidance and adaptation (Tränkner et al., 2018). It also reduces the photosynthetic efficiency (Jin et al., 2011), consequently affecting carbon partitioning to wood production, influencing the plant’s anatomical composition (Epron et al., 2012). Moreover, maximum growth can be reached with concomitant application of Na and K, in addition to improving stomatal conductance (*g*_*s*_) and mitigating the anatomical and biochemical deficiencies of plants caused by low K availability (Battie-Laclau et al., 2013; Mateus et al., 2019).

A comprehensive literature indicates the benefits of Na supply to plants (Hampe and Marschner, 1982; Subbarao et al., 1999; Martínez et al., 2005; Idowu and Aduayi, 2006; Ivahupa et al., 2006; Ma et al., 2011; Wakeel et al., 2011; Kronzucker et al., 2013; Erel et al., 2014; Krishnasamy et al., 2014; Pi et al., 2014), as also in drought adaptations of halophyte plants (Lv et al., 2012; Yue et al., 2012; Xi et al., 2018). Non-halophytic plants, such as *Eucalyptus*, although salt-sensitive (Pardo and Quintero, 2002), are also able to utilize Na to some extent (Subbarao et al., 2003; Mateus et al., 2019). Depending on the species and the levels applied, the Na supply may be toxic for plants (Kronzucker et al., 2013), which in turn demands more attention with regard to using Na in fertilizing non-halophytes in order to fulfill plants’ nutritional requirements under K deficiency (Mateus et al., 2019). Plants can behave differently under nutritional stress conditions and vary in nutritional efficiency (Pita-Barbosa et al., 2016), which allows some species to grow more at the highest levels of Na (Subbarao et al., 2003). However, despite the great variety of studies involving nutrient application, plant growth, and water deficit (Müller et al., 2017), studies involving K and Na use efficiency of different species and water regimes are still scarce. Thus, this study aimed to evaluate the partial replacement of K by Na and its impacts on water use and K use efficiency in three useful *Eucalyptus* species under different water conditions, investigating to what extent Na can substitute K and attenuate the effects of drought.

## Materials and Methods

### Experimental Design and Growth Conditions

The experiment was carried out in a greenhouse at the Center for Nuclear Energy in Agriculture (CENA-USP) in Piracicaba, São Paulo State, Brazil, from July to November 2018. Plants were grown at temperatures between 18 and 32°C (mean of 25°C) with an average relative humidity of 65%. Three *Eucalyptus* species with different levels of drought tolerance (*Eucalyptus saligna* Sm., drought sensitive; *Eucalyptus urophylla* S.T. Blake, moderate tolerance; and *Eucalyptus camaldulensi*s Dehn., drought tolerant) (Gonçalves et al., 2017) of approximately 90 days old and 30 cm in height, germinated from seeds obtained from the Institute of Forest Research and Studies (IPEF, Brazil), were transplanted into individual plastic pots (5 kg) containing Oxisol soil (16% clay, 5% silt, and 79% sand), collected from the top layer at the Itatinga Experimental Station, Itatinga, São Paulo State, Brazil. The physiochemical characteristics were: pH = 4.2; organic matter = 25 g dm^–3^; P = 4 mg dm^–3^; K = 0.3 mmol_*c*_ dm^–3^; Ca = 1 mmol_*c*_ dm^–3^; Mg = 1 mmol_*c*_ dm^–3^; H + Al = 25 mmol_*c*_ dm^–3^; Al = 3 mmol_*c*_ dm^–3^; B = 0.14 mg dm^–3^; Cu = 0.6 mg dm^–3^; Fe = 33 mg dm^–3^; Mn = 0.8 mg dm^–3^; and Zn = 0.8 mg dm^–3^.

Based on the soil K critical level (<1.20 mmol_*c*_ dm^–3^ of K) for *Eucalyptus* to respond to potassium fertilization, K was replaced by Na (as NaCl), representing substitutions of 0/100, 25/75, 50/50, 75/25, and 100/0 (percentage of Na/percentage of K) for 120 days. Thus, the treatments consisted of five combinations of Na and K application rates (0/0.90, 0.22/0.67, 0.44/0.44, 0.67/0.22, and 0.90/0 mmol_*c*_ dm^–3^ of Na/millimoles of charge per cubic decimeter of K), which, when added to the soil K content, reached the soil K critical level (1.20 mmol_*c*_ dm^–3^ of K). The rates 0 and 0.90 mmol_*c*_ dm^–3^ of Na represented the control (solely K supplied plants) and the K deficiency treatments, respectively. We would like to emphasize that the Na rates employed in studies regarding salinity-induced stress in plants are higher than those used hereby. For instance, Zhang et al. (2016) investigated salinity-induced stress on wheat seedlings’ growth in nutrient solution by adding a NaCl rate of 150 mM, while Quais et al. (2020) characterized the mechanisms of the underlying interactions between rice plants and brown planthopper under salinity stress (100 mM salinity level). According to Madsen and Mulligan (2006), the emergence of *Eucalyptus citriodora*, *E. camaldulensis*, *Eucalyptus populnea*, and *Acacia salicina* was substantially reduced by adding 100 mM of NaCl, while the survival of established plants was reduced only at 300 and 400 mM of NaCl.

In addition to the treatments with K and Na, all samples received the following complementary fertilization: 135 mg dm^–3^ of N plus 20 mg dm^–3^ of N in coverage at 2 months after the onset of the treatments, 300 mg kg^–1^ of P, 92 mg kg^–1^ of Ca, 7.2 mg kg^–1^ of Mg (reaching 7 mmol_*c*_ dm^–3^ in a Ca^+2^/Mg^+2^ ratio of 4:1), 45 mg kg^–1^ of S, 0.82 mg kg^–1^ of B, 4.0 mg kg^–1^ of Zn, 3.66 mg kg^–1^ of Mn, 1.55 mg kg^–1^ of Fe, 1.39 mg kg^–1^ Cu, and 0.20 mg kg^–1^ of Mo, facilitating adequate plant development (Novais et al., 1991). The sources of the elements used were as follows: (NH_4_)H_2_PO_4_, CaCO_3_, MgCO_3_, elementary S, CuSO_4_.5H_2_O, ZnSO_4_.7H_2_O, FeSO_4_.7H_2_O, H_3_BO_3_, MnSO_4_.H_2_O, and MoO_3_.H_2_O.

The plants were exposed to two water conditions starting 60 days after the onset of the treatments: well-watered (WW) and water stress (WS) conditions, simulating adequate water availability and drought conditions, respectively. The soil relative water content (SRWC) of both water conditions was controlled daily by the gravimetric method using irrigation with deionized water up to 80 and 35% of the field capacity under WW and WS, respectively. Weighing and watering were conducted on a daily basis at dusk until the pots reached their corresponding target-adapted SRWC (Equation 1) (Xu et al., 2009); every 15 days, two plants were harvested and weighed to maintain the desired SRWC in the pots.

(1) $$\mathrm{SRWC}\left(\%\right):\frac{\left(\mathrm{Wtotal}-\mathrm{Wpot}-\mathrm{DWsoil}-\mathrm{SFW}\right)}{\left(\mathrm{WFC}-\mathrm{Wpot}-\mathrm{DWsoil}-\mathrm{SFW}\right)}*100$$

where *W*_*to*__*tal*_ is the current soil total weight (pot + soil + plant + water), *W*_*pot*_ is the weight of the empty pot, DW_*soil*_ is the dry soil weight, SFW is the fresh weight of two plants harvested every 15 days, and WFC is the soil weight at field capacity (soil + pot + water).

The experiment was performed in randomized blocks, with four replications in a 5 × 2 factorial design (five rates of K replacement by Na and two water conditions) for each *Eucalyptus* species, totaling 120 experimental units with one plant each.

### Leaf Gas Exchange and Leaf Water Potential

The youngest fully expanded leaf of each plant was used to evaluate gas exchange in the morning (from 9 a.m. to 11 a.m.) using an infrared gas analyzer (IRGA, Li-6400XT, LICOR Inc., Lincoln, NE, United States) at environmental humidity and temperature. The external CO_2_ concentration (*C*_*a*_) was fixed at 400 μmol and the photosynthetically active radiation (PAR) flux density at 1,200 μmol m^–2^ s^–1^. Photosynthesis (*A*), stomatal conductance (*g*_*s*_), and transpiration (*E*) were measured (Mateus et al., 2019). Mean leaf temperature during the measurement was 30°C. WUE (in grams dry matter per kilogram H_2_O^–1^) was calculated by dividing the total dry matter (TDM) value (belowground plus aboveground) by water consumption (WC) throughout the experiment (Martin and Thorstenson, 1988), which was obtained by calculating the daily weight reduction due to transpiration (Equation 2).

(2) WC:WFC-(Wsoil-Wpot-DWsoil-SFW-Sevap)

where *S*_*evap*_ is the soil evaporation from the mean weight loss of four plantless pots.

In the same leaves, the predawn (3 a.m.) and noon (12 p.m.) leaf water potentials (*Ψ*w_*PD*_ and *Ψ*w_*N*_, respectively) were also measured using a Scholander pressure chamber (Turner, 1981). All evaluations were realized prior to harvesting.

### Stomatal Density and Leaf Area

Stomatal density (Std, stomates per square millimeter) was calculated using the two youngest fully expanded leaves per plant (Mateus et al., 2019) on abaxial and adaxial surfaces, applying the software package ImageJ^1^. Complementary micrograph material of Std was obtained by scanning electron microscopy (JEOL JSM-IT300 LV, Tokyo-Japan) at 20 kV, and digital images were recorded (Lavres et al., 2019).

Plants were harvested 120 days after the onset of the treatments, and their leaves, stems, branches, and roots were separated. Leaf area (LA) was obtained by passing all leaves through a leaf area integrator (LI-3100).

### Dry Matter Production and Mineral Element Analysis

After drying in a forced air ventilation oven at 60°C for 72 h, each plant part was weighed to determine dry matter. Subsequently, the plant material was ground in a Wiley-type mill and forwarded to nitric–perchloric digestion (Malavolta et al., 1997) to quantify K and Na by inductively coupled plasma optical emission spectrometry (ICP-OES; iCAP 7000 Series, Thermo Fisher Scientific, Waltham, United States). Based on the leaf K and Na concentrations, we calculated the K/Na ratio, which was correlated with the estimated rate of maximum dry matter production (critical level of 90% maximum yield) of each genotype, obtained by equaling the equation to zero. The accumulations of K and Na were obtained by multiplying the concentration of each element in the tissue by the dry matter production of the respective tissue (root, stems, and leaves) and used to determine the use efficiency (UE, in grams per milligram) (Siddiqi and Glass, 1981) according to Equation 3.

(3) $$\mathrm{UE}:\frac{\left(\mathrm{plant}\,\mathrm{dry}\,\mathrm{matter}\right)\,2\,\left(\text{g}\right)}{\mathrm{nutrient}\,\mathrm{in}\,\mathrm{plant}\,\left(\mathrm{mg}\right)}$$

where nutrient refers to K or Na accumulation.

### Leaf Carbon Isotope Composition (δ^13^C‰)

The same samples used for leaf dry matter determination were also used to assess the carbon isotope composition, determined using a mass spectrometer (ANCA-GSL Hydra 20-20 model, SERCON Co., Crewe, GBR) coupled to a C automatic analyzer (Barrie and Prosser, 1996), and the isotope values (in per mill) were calculated *via* Equation 4 (Farquhar and Sharkey, 1982).

(4) $$\delta \,13\,C\,\left(‰\right):\left(\frac{\text{Rsample}}{\mathrm{Rpdb}}-1\right)*1000$$

where *R* is the ratio of ^13^C/^12^C. The reference material is the Vienna Pee Dee Belemnite (PDB).

### Statistical Analyses

Data were analyzed by the *F* test (*p* < 0.05), and significant differences among means were determined *via* Tukey’s *post-hoc* test (*p* < 0.05) to compare the WW and WS conditions. The significant effects of Na application were described by linear, quadratic, and square root regression models, in which the significant model (*p* < 0.05) with the highest determination coefficient (*R*^2^) was selected. The original data were standardized to be analyzed by principal component analysis (PCA) and cluster analysis, correlating the measured variables in each genotype and water condition. In cluster analysis, the treatments were grouped into functional units by their similarity; for the PCA, we used the treatments with Na supply for the first two main components (PC1 and PC2).

Statistical analyses were performed using the software packages SAS version 9.1 (SAS Institute Inc, 2004) and R version 3.5.1 (R Development Core Team, 2018). Data variability was indicated with standard error and shown graphically using SigmaPlot 11.0 (Systat Software Inc., San Jose, CA, United States).

## Results

### Adaxial and Abaxial Stomatal Density

**FIGURE 1:**
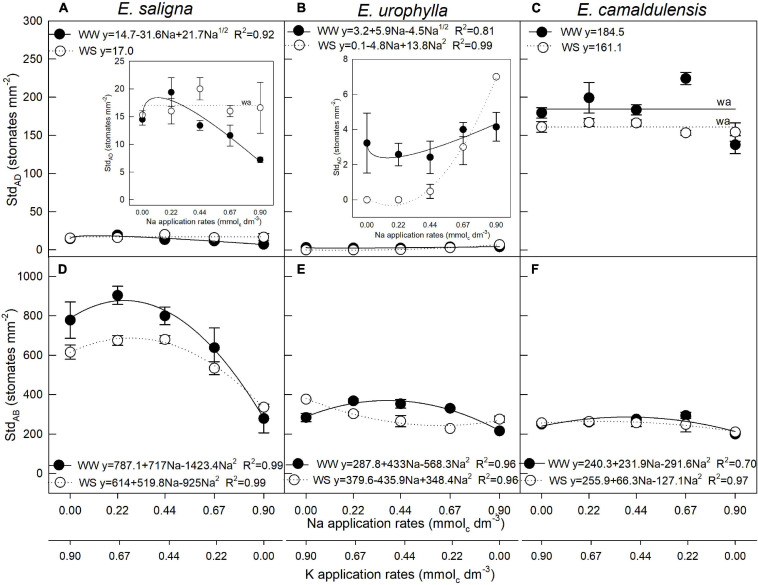
Stomatal density of the adaxial (Std_*AD*_) **(A–C)** and abaxial (Std_*AB*_) **(D–F)** leaf surfaces of *Eucalyptus saligna* **(A,D)**, *Eucalyptus urophylla* **(B,E)**, and *Eucalyptus camaldulensis* **(C,F)** seedlings under K partial replacement by Na in well-watered (WW) and water-stressed (WS) conditions. *Vertical bars* indicate standard errors among blocks (*n* = 4). The adjustment model is indicated by not significant (*ns*) and without suitable adjustment (*wa*).

The leaves of *E. saligna* and *E. urophylla* were hypostomatic, occurring mainly on the abaxial surface, with lower than 25 stomates per square millimeter. In *E. camaldulensis*, however, the leaves were amphistomatous, with stomates occurring on both surfaces. Adaxial stomatal density (Std_*AD*_) was influenced by Na, WS, and Na^∗^WS in *E. saligna*, *E. urophylla*, and *E. camaldulensis* (Figures 1A–C). Abaxial stomatal density (Std_*AB*_) was influenced by Na and WS in *E. saligna* and *E. urophylla*, whereas in *E. camaldulensis*, it was affected by Na application (Figures 1D–F). *E. saligna* and *E. camaldulensis* seedlings grown under WW and WS conditions showed higher Std_*AD*_ and Std_*AB*_ levels with partial K replacement by Na. Additionally, the highest Na rate (0.9 mmol_*c*_ dm^–3^ of Na) led to decreased Std_*AB*_ levels by 50, 30, and 20% in *E. saligna*, *E. urophylla*, and *E. camaldulensis*, respectively. Water stress also decreased the mean Std_*AB*_ by 15 and 10% in *E. saligna* and *E. urophylla*, respectively, irrespective of the Na rate.

### Leaf Gas Exchange

**FIGURE 2:**
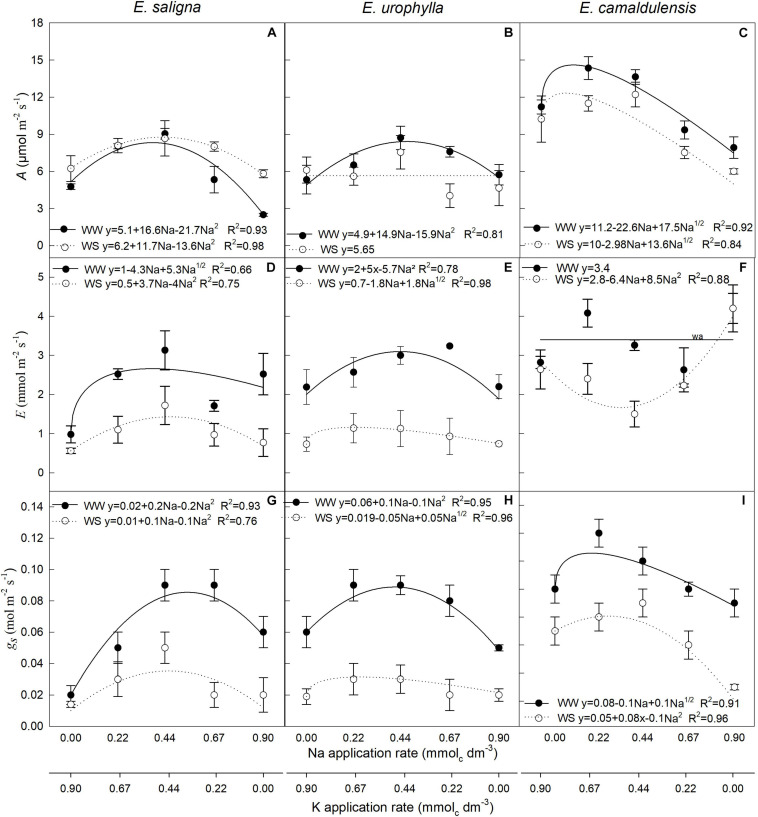
Photosynthetic rate (*A*) **(A–C)**, leaf transpiration rate (*E*) **(D–F)**, and stomatal conductance (*g*_*s*_) **(G–I)** in the leaves of *Eucalyptus saligna* **(A,D,G)**, *Eucalyptus urophylla* **(B,E,H)**, and *Eucalyptus camaldulensis* **(C,F,I)** seedlings under K partial replacement by Na in well-watered (WW) and water-stressed (WS) conditions. *Vertical bars* indicate standard errors between blocks (*n* = 4). The without suitable adjustment of a model is indicated by *wa*.

Parameters *A* (assimilation rate; Figures 2A–C), *E* (transpiration rate; Figures 2D–F), and *g*_*s*_ (Figures 2G–I) were influenced by Na and WS in all species. Partial K replacement by Na (up to 0.44 mmol_*c*_ dm^–3^) increased *A* up to 55, 50, and 20% in *E. saligna*, *E*. *urophylla*, and *E*. *camaldulensis*, respectively, when compared to the control (0 mmol_*c*_ dm^–3^ of Na). Meanwhile, the K-deficient plants of all genotypes had lower *A*. Compared to the control, the intermediary rates of Na also increased *E* up to 200 and 50% in *E. saligna* and *E*. *urophylla*, respectively, under both water conditions, and 40% in *E. camaldulensis* under WW. Against the other genotypes, *E* decreased until the intermediary Na rates for *E. camaldulensis* under WS conditions. The K-deficient plants had higher *E*, except for *E*. *urophylla* under WW. The *g*_*s*_ increased with partial K replacement by Na up to 250% in *E. saligna* and to 50% in *E*. *urophylla* and *E. camaldulensis* under both water conditions compared to the control. K-deficient plants had significantly lower *g*_*s*_ in *E*. *urophylla* under WW and *E. camaldulensis* under WS. Considering the mean of all rates, drought increased *A* by 20% in *E. saligna* and decreased its values by 15% in *E. urophylla* and *E*. *camaldulensis*; it also reduced the *E* values by 45, 35, and 75% and the *g*_*s*_ values by 50, 30, and 55%, in *E. saligna*, *E. urophylla*, and *E. camaldulensis*, respectively, compared to those under WW.

### Leaf Carbon Isotope Composition (δ^13^C‰)

**FIGURE 3:**
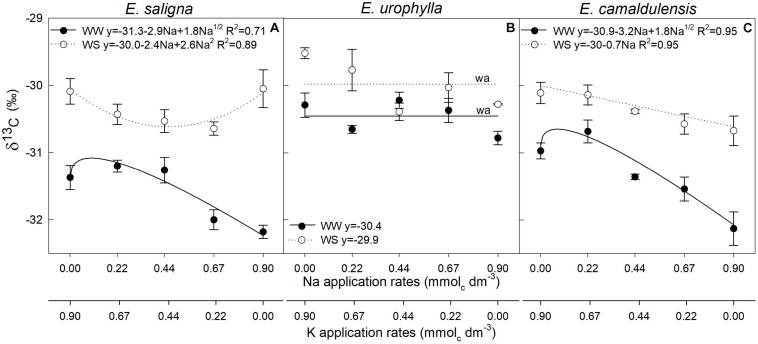
Leaf Carbon Isotope Composition (δ^13^C‰) of *Eucalyptus saligna* **(A)**, *Eucalyptus urophylla* **(B)**, and *Eucalyptus camaldulensis* **(C)** seedlings under K partial replacement by Na in well-watered (WW) and water-stressed (WS) conditions. *Vertical bars* indicate standard errors among blocks (*n* = 4). The adjustment model is indicated by not significant (*ns*) and without suitable adjustment (*wa*).

Factors Na, WS, and Na^∗^WS significantly influenced the leaf carbon isotopic compositions (δ^13^C‰) of *E. saligna* and *E*. *urophylla*, whereas for *E. camaldulensis*, it was affected by Na and WS (Figures 3A–C). Under WW, for *E. saligna* and *E*. *camaldulensis*, the δ^13^C‰ increased with lower Na application rates (0.22 mmol_*c*_ dm^–3^ of Na) and decreased with higher Na rates. In contrast, the values of δ^13^C‰ for *E. urophylla* were reduced at lower rates (0.22 mmol_*c*_ dm^–3^ of Na). The lowest δ^13^C‰ values were observed in K-deficient plants (0.9 mmol_*c*_ dm^–3^ of Na) of *E. urophylla* and *E. camaldulensis* under both water conditions and in the intermediate Na rates (0.44 and 0.67 mmol_*c*_ dm^–3^ of Na) of *E. saligna* under WS. Drought stress increased the δ^13^C‰ values of all species when compared to WW.

### Predawn and Noon Leaf Water Potentials

**FIGURE 4:**
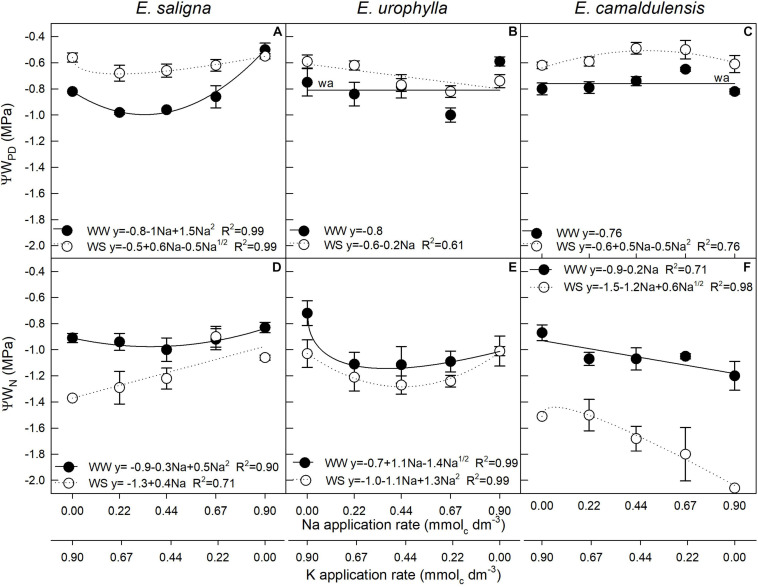
Predawn (*Ψ*w_*PD*_) **(A–C)** and noon (*Ψ*w_*N*_) **(D–F)** leaf water potentials of *Eucalyptus saligna* **(A,D)**, *Eucalyptus urophylla* **(B,E)**, and *Eucalyptus camaldulensis* **(C,F)** seedlings under K partial replacement by Na in well-watered (WW) and water-stressed (WS) conditions. *Vertical bars* indicate standard errors between blocks (*n* = 4).

In *E*. *saligna*, both predawn (*Ψ*w_*PD*_) and noon (*Ψ*w_*N*_) leaf water potentials were affected by Na, WS, and Na^∗^WS (Figures 4A,D), while in *E*. *urophylla* (Figures 4B,E) and *E*. *camaldulensis* (Figures 4C,F), these were affected by Na and WS. The lowest *Ψ*w_*PD*_ and *Ψ*w_*N*_ values were found at low to intermediate Na rates (0.22 and 0.44 mmol_*c*_ dm^–3^) in *E*. *saligna* as well as for *E*. *urophylla* under both water conditions, except the *Ψ*w_*N*_ of *E*. *saligna* under WW, which increased linearly with Na application. Otherwise, the *Ψ*w_*PD*_ of *E. camaldulensis* increased up to 0.67 mmol_*c*_ dm^–3^ in both conditions, while the *Ψ*w_*N*_ decreased with increasing Na application rates. The *Ψ*w_*PD*_ values of all genotypes were lower under WS than under WW, while the opposite was found in *Ψ*w_*N*_ values since the WS conditions decreased up to 25, 10, and 55% for *E. saligna*, *E. urophylla*, and *E. camaldulensis*, respectively, considering the mean of all Na application rates.

### Total Dry Matter Yield

The TDM of all genotypes was affected by Na, WS, and Na^∗^WS (Table 1). Partial K replacement by Na increased the TDM of all genotypes under both conditions, except in *E. saligna* under WS, which had a higher TDM than the control (0 mmol_*c*_ dm^–3^ of Na), decreasing linearly with higher Na rates. The K-deficient plants (0.9 mmol_*c*_ dm^–3^ of Na) had lower TDM levels by 35% under both water conditions compared to the control. Under WW, the maximum TDM (critical level) was reached with 0.048 mmol_*c*_ dm^–3^ Na and 0.852 mmol_*c*_ dm^–3^ K, corresponding to 5.3% substitution and 48.9 g per plant.

**TABLE 1:** Mean values (±standard errors, *n* = 4) of total dry matter (TDM) production, leaf area, and long-term water use efficiencies (WUE_*L*_) in the leaves of *Eucalyptus saligna*, *Eucalyptus urophylla*, and *Eucalyptus camaldulensis* seedlings under K partial replacement by Na in well-watered (WW) and water-stressed (WS) conditions.

**Na application rate (mmol dm^–3^)**	**TDM (g per plant)**		**Leaf area (m^2^ per plant)**		**WUE (plant dry matter kg^–1^ H_2_O)**	
			
	**WW**	**WS**	**WW**	**WS**	**WW**	**WS**
*E. saligna*	*, **, ***		*, **,***		*, **,***	
0	50.54 ± 3.96	49.1 ± 5.00	0.18 ± 0.009	0.12 ± 0.002	1.65 ± 0.050	2.63 ± 0.105
0.22	53.8 ± 9.30	40.45 ± 2.00	0.13 ± 0.007	0.12 ± 0.003	1.76 ± 0.050	1.37 ± 0.090
0.44	42.23 ± 4.50	39.55 ± 3.90	0.14 ± 0.013	0.13 ± 0.005	1.42 ± 0.135	1.74 ± 0.250
0.67	37.6 ± 8.40	39.96 ± 2.10	0.13 ± 0.009	0.13 ± 0.008	1.30 ± 0.090	1.92 ± 0.075
0.9	32.42 ± 60	31.5 ± 4.50	0.10 ± 0.010	0.10 ± 0.003	1.19 ± 0.025	2.0 ± 0.270
Model	*y* = 51 - 43.25Na + 20Na^0.5^	*y* = 45.6 - 14.1Na	*y* = 0.27 + 0.28Na - 0.42Na^0.5^	wa	*y* = 1.66 - 1.11Na + 0.51Na^0.5^	*y* = 2.6 + 3.6Na - 3.94Na^0.5^
*R*^2^	0.93	0.6	0.94		0.89	0.9
*E. urophylla*	*, **, ***		*, **		*, **	
0	71.65 ± 1.30	56.2 ± 3.85	0.18 ± 0.010	0.13 ± 0.020	2.22 ± 0.080	2.69 ± 0.170
0.22	73.52 ± 3.00	61.6 ± 4.15	0.20 ± 0.020	0.14 ± 0.003	2.25 ± 0.130	2.62 ± 0.205
0.44	65.2 ± 0.22	52.0 ± 2.50	0.29 ± 0.010	0.16 ± 0.020	2.23 ± 0.155	2.61 ± 0.155
0.67	46.7 ± 3.95	47.4 ± 4.55	0.18 ± 0.030	0.14 ± 0.020	1.54 ± 0.060	2.45 ± 0.255
0.9	36.6 ± 4.15	39.0 ± 2.00	0.17 ± 0.030	0.10 ± 0.004	1.33 ± 0.125	2.41 ± 0.055
Model	*y* = 71.7 - 89.4Na + 46.4Na^0.5^	*y* = 56.1 - 55Na + 33.7Na^0.5^	wa	*y* = 0.12 + 0.18Na - 0.22Na^2^	*y* = 2.19 + 0.12Na - 1.26Na^2^	*y* = 2.71 - 0.24Na - 0.11Na^2^
*R*^2^	0.98	0.96		0.93	0.84	0.92
*E. camaldulensis*	*, **, ***		*, **		*, **,***	
0	69.5 ± 5.76	50.3 ± 5.00	0.15 ± 0.003	0.10 ± 0.009	2.20 ± 0.040	2.88 ± 0.030
0.22	74.7 ± 5.85	51.3 ± 3.00	0.16 ± 0.010	0.10 ± 0.015	2.35 ± 0.110	2.98 ± 0.140
0.44	68.08 ± 4.10	47.8 ± 5.55	0.16 ± 0.020	0.10 ± 0.002	2.05 ± 0.040	2.96 ± 0.060
0.67	65.25 ± 5.74	42.7 ± 4.00	0.14 ± 0.008	0.08 ± 0.004	2.30 ± 0.030	2.40 ± 0.020
0.9	31 ± 7.49	32.8 ± 0.98	0.10 ± 0.010	0.06 ± 0.020	1.52 ± 0.305	1.62 ± 0.195
Model	*y* = 67.1 + 69.9Na - 116.7Na^2^	*y* = 51.3 + 6Na - 29.2Na^2^	*y* = 0.14 + 0.08Na - 0.14Na^2^	*y* = 0.1 + 0.02Na - 0.07Na^2^	*y* = 2.18 + 1Na - 1.82Na^2^	*y* = 2.87 + 1.27Na - 2.98Na^2^
*R*^2^	0.83	0.99	0.99	0.98	0.72	0.98

For each parameter, *, **, and *** indicate the statistical influence (F test with a significance threshold of p < 0.05) of Na rates, WS, and WS × Na, respectively. The adjustment model is indicated by without suitable adjustment (wa).

In *E. urophylla*, a higher TDM level was found in plants with low Na rates (0.22 mmol_*c*_ dm^–3^) at both water conditions, whereas K-deficient plants had decreased TDM by 50 and 30% than the control treatment in WW and WS, respectively. The estimated Na rate to give the maximum TDM was 0.06 mmol_*c*_ dm^–3^ of Na and 0.84 mmol_*c*_ dm^–3^ of K, which corresponded to 6.7% of substitution reaching 69.9 g per plant. Under WS, the estimated Na rate was 0.085 mmol_*c*_ dm^–3^ and 0.815 mmol_*c*_ dm^–3^ of K, corresponding to 9.3% substitution and 55.13 g per plant.

In contrast to the other genotypes, the higher TDM values of *E*. *camaldulensis* were observed up to an Na rate of 0.44 mmol_*c*_ dm^–3^ under both water conditions, which means a substitution of K by Na around 50%. The K-deficient plants had lower TDM by 55 and 35% than the control treatment under the WW and WS conditions, respectively. The rates estimated to obtain the maximum TDM under WW was 0.27 mmol dm^–3^ of Na and 0.63 mmol_*c*_ dm^–3^ of K, corresponding to a substitution level of 30% and 69.81 g per plant. Under WS, the estimated rates were 0.09 mmol_*c*_ dm^–3^ of Na and 0.81 mmol_*c*_ dm^–3^ of K, corresponding to 10% K replacement by Na and 46.4 g per plant.

### Leaf Area

Leaf area (LA) was influenced by Na, WS, and Na^∗^WS in *E. saligna* (Table 1). Under WW, LA decreased up to 30% with higher Na application, while under WS, an increase around 10% occurred with intermediary rates of Na (0.44 and 0.67 mmol_*c*_ dm^–3^) when compared to the control (0 mmol_*c*_ dm^–3^ of Na). The LA of *E. urophylla* and *E. camaldulensis* was affected by Na and WS. Low to intermediary Na rates (0.22 and 0.44 mmol_*c*_ dm^–3^) increased the LA of *E. urophylla* by up to 60 and 25% compared to the control under WW and WS, respectively. In *E. camaldulensis*, these Na rates increased the LA by 6% compared to the control under WW. Otherwise, K-deficient plants (0.9 mmol_*c*_ dm^–3^ of Na) significantly decreased the LA up to 40% in all genotypes compared to the control. Water stress decreased the LA by 15, 35, and 36% in *E. saligna*, *E. urophylla*, and *E. camaldulensis*, respectively, according to the mean of all Na rates.

### Water Use Efficiency

Water use efficiency (WUE) was influenced by Na, WS, and Na^∗^WS in *E. saligna* and *E. camaldulensis*, whereas in *E. urophylla*, it was only affected by Na and WS (Table 1). In *E. saligna* and *E. urophylla* under WW and in *E. camaldulensis* under both conditions, low K replacement by Na increased the WUE and decreased it in higher Na rates. Otherwise, plants of *E. saligna* and *E. urophylla* under WS decreased the WUE due to Na supply. Drought stress increased the WUE by 33, 35, and 17% in *E. saligna*, *E. urophylla*, and *E. camaldulensis*, respectively, irrespective of the Na rate. In addition, the mean WUE was higher in *E. camaldulensis* (drought tolerant), followed by *E. urophylla* (moderate tolerance) and *E*. *saligna* (drought sensitive) in both water conditions.

### K and Na Leaf Concentrations and Efficiency of Use

**FIGURE 5:**
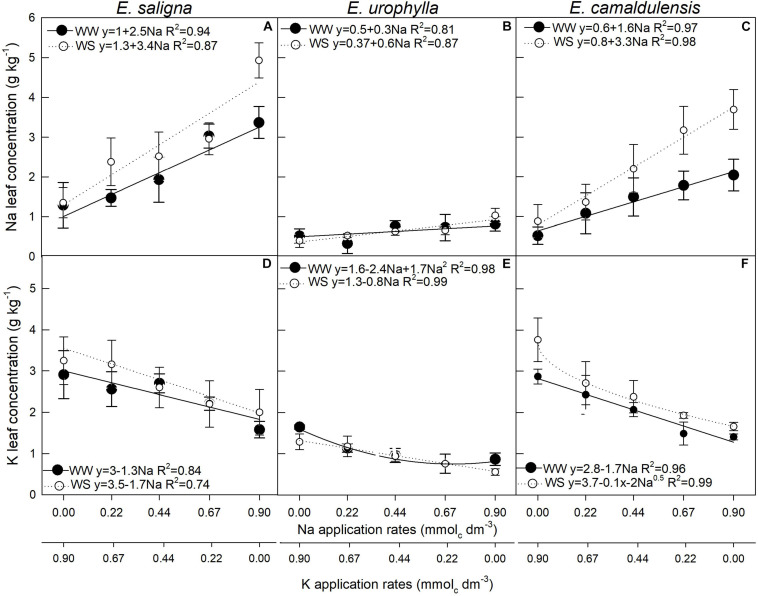
Leaf Na **(A–C)** and leaf K **(D–F)** concentrations of *Eucalyptus saligna* **(A,D)**, *Eucalyptus urophylla* **(B,E)**, and *Eucalyptus camaldulensis* **(C,F)** seedlings under K partial replacement by Na in well-watered (WW) and water-stressed (WS) conditions. *Vertical bars* indicate standard errors between blocks (*n* = 4).

The Na leaf concentration [Na] was influenced by Na and WS in *E. saligna* (Figure 5A), *E*. *urophylla* (Figure 5B), and *E*. *camaldulensis* (Figure 5C), While the K leaf concentration [K] was affected by Na, WS, and Na^∗^WS (Figure 5D) in *E*. *saligna*, *E*. *urophylla* (Figure 5E), and *E*. *camaldulensis* (Figure 5F). This affected only the Na rates and WS. Overall, K decreased and Na increased with increasing Na rates. In addition, the concentration levels were higher in plants under WW than in WS. In *E. saligna* under WW, the replacement of 5.3%, corresponding to 90% of TDM and reaching rates of 0.041 and 0.85 mmol_*c*_ dm^–3^ of Na and K, respectively, decreased the K by 0.06 g kg^–1^, while Na increased by 0.12 g kg^–1^ compared to the application of only K, increasing plant growth. Furthermore, K of 2.9 g kg^–1^ was still above the critical level of K, without symptoms of deficiency. *E. urophylla* under WW with 6.7% of K replacement by Na reached the rates 0.06 and 0.83 mmol_*c*_ dm^–3^ of Na and K, respectively; K decreased by 0.13 g kg^–1^, while Na increased by 0.018 g kg^–1^ compared to the application of only K. Under WS, 9.3% of K replacement by Na reached the rates 0.085 and 0.815 mmol_*c*_ dm^–3^ of Na and K, respectively; K decreased by 0.07 g kg^–1^, while Na increased by 0.052 g kg^–1^ compared to the application of only K. Conversely, *E. camaldulensis* under WW, with 30% of K replacement by Na, reached the rates 0.27 and 0.63 mmol_*c*_ dm^–3^ Na and K, respectively; K decreased by 0.46 g kg^–1^, while Na increased by 0.44 g kg^–1^ compared to the application of only K.

Additionally, the leaf K/Na ratios decreased with increasing Na levels. According to the estimated rate of Na to achieve the maximum TDM of *E. saligna*, *E*. *urophylla*, and *E. camaldulensis*, the optimal leaf K/Na ratios were 1.7, 2.9, and 2.2, respectively, under WW and 2.6, 2.2, and 3.2, respectively, under WS (Table 2). UE_*K*_ was affected by Na and WS, increasing up to 30% mainly with low K replacement by Na, while UE_*Na*_ was affected by Na, WS, and Na^∗^WS (Table 2), decreasing up to 70% with higher K replacement by Na. Water stress decreased the UE of both K and Na compared to WW.

**TABLE 2:** Mean values (±standard errors, *n* = 4) of leaf K/Na ratio and K and Na use efficiency of *Eucalyptus saligna*, *Eucalyptus urophylla*, and *Eucalyptus camaldulensis* seedlings under K partial replacement by Na in well-watered (WW) and water-stressed (WS) conditions.

**Na application rate (mmol dm^–3^)**	**Leaf K/Na ratio**		**Use efficiency (g^2^ mg^–1^)**			
		
			**K**		**Na**	
	**WW**	**WS**	**WW**	**WS**	**WW**	**WS**
*E. saligna*	*, ***		*, **		*, **, ***	
0	2.14 ± 0.12	2.97 ± 0.31	20.8 ± 2.8	21.7 ± 1.5	54.9 ± 6.7	59 ± 4.5
0.22	1.54 ± 0.17	1.49 ± 0.14	28.5 ± 3.0	19.2 ± 2.2	51.7 ± 1.4	29.4 ± 4.3
0.44	1.32 ± 0.22	1.05 ± 0.07	27.3 ± 0.9	25.9 ± 3.2	32.7 ± 2.1	24.2 ± 4.7
0.67	0.81 ± 0.13	0.80 ± 0.11	33.5 ± 1.5	29.2 ± 2.7	23.5 ± 2.6	21.7 ± 2.6
0.9	0.44 ± 0.09	0.41 ± 0.03	30.1 ± 3.6	22.7 ± 3.3	14.5 ± 2.4	9.5 ± 2.3
Model	*y* = 2 - 1.8Na	*y* = 2.9 + 0.86Na - 3.45Na^0.5^	*y* = 20.8 - 8.5Na + 19Na^0.5^	wa	*y* = 55 - 59.4Na + 11Na^0.5^	*y* = 58.4 + 15.7Na - 63.2Na^0.5^
*R*^2^	0.98	0.99	0.82		0.96	0.97
*E. urophylla*	*		*, **		*, **, ***	
0	3.74 ± 0.46	3.00 ± 0.49	46.5 ± 2.5	38.7 ± 4.4	119.2 ± 5.1	87 ± 6.5
0.22	2.41 ± 0.27	1.95 ± 0.27	60.87 ± 5.0	45.6 ± 5.7	82.7 ± 4.0	63.7 ± 5.7
0.44	1.54 ± 0.12	1.25 ± 0.10	47.14 ± 4.2	41.74 ± 2.1	63.5 ± 0.5	45.3 ± 0.7
0.67	0.7 ± 0.04	0.79 ± 0.06	46.48 ± 0.7	40.01 ± 2.3	33.8 ± 4.7	36 ± 3
0.9	0.4 ± 0.04	0.50 ± 0.03	37.8 ± 4.5	33.82 ± 1.9	17.8 ± 3.9	17.8 ± 1.4
Model	*y* = 3.7 - 6.5Na + 3Na^2^	*y* = 2.98 - 5Na + 2.6Na^2^	*y* = 47.1 - 65.78Na + 51.7Na^0.5^	*y* = 38.72 - 38.62Na + 31.87Na^0.5^	*y* = 113.3 - 111.75Na	*y* = 82.86 - 73.6Na
*R*^2^	0.99	0.99	0.83	0.96	0.98	0.97
*E. camaldulensis*	*, **		*, **,		*, **, ***	
0	6.17 ± 0.41	4.62 ± 1.04	51.5 ± 5.0	21.8 ± 0.3	141.84 ± 5.0	63.4 ± 6.3
0.22	3.1 ± 0.22	2.79 ± 0.09	53.4 ± 4.7	26.8 ± 0.1	90.5 ± 4.7	55.8 ± 4.2
0.44	1.26 ± 0.10	0.96 ± 0.16	52.4 ± 5.0	31.7 ± 1.0	67.33 ± 5.0	48.8 ± 6.4
0.67	0.84 ± 0.06	0.75 ± 0.07	73.2 ± 4.2	30.1 ± 0.2	68.17 ± 4.2	26.2 ± 1.7
0.9	0.75 ± 0.20	0.52 ± 0.11	32.1 ± 4.8	28.5 ± 1.2	41.48 ± 4.8	17.2 ± 0.6
Model	*y* = 6 - 15.3Na + 10.6Na^2^	*y* = 4.6 - 10.8Na + 6.9Na^2^	*y* = 5.2 - 2.33Na	*y* = 7.98 + 0.29Na - 2.15Na^0.5^	*y* = 1.93 + 2.93Na	*y* = 2.2 + 1.53Na + 8.6Na^2^
*R*^2^	0.99	0.98	0.77	0.99	0.74	0.99

For each parameter, *, **, and *** indicate the statistical influence (F test with a significance threshold of p < 0.05) of Na rates, WS, and WS × Na, respectively. The adjustment model is indicated by without suitable adjustment (wa).

### Characterization Among Genotypes

**FIGURE 6:**
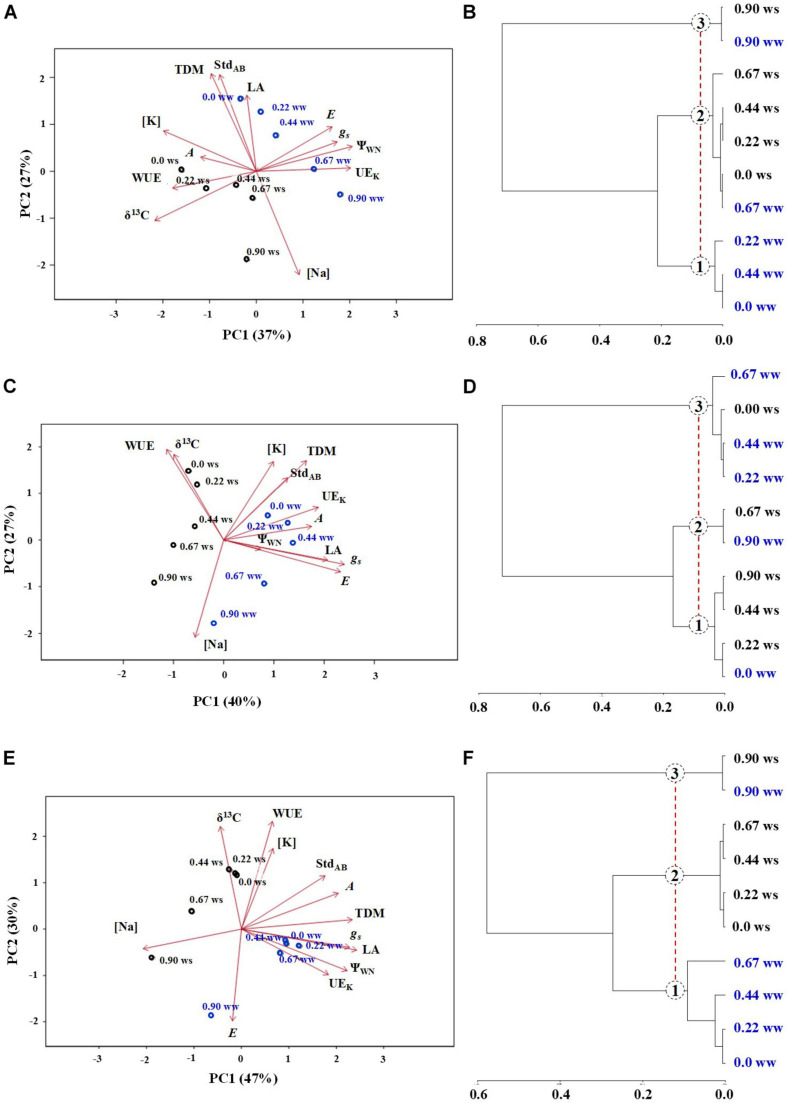
Principal component analysis (PCA) **(A,C,E)** and hierarchical cluster analyses **(B,D,F)** of *Eucalyptus saligna* **(A,B)**, *Eucalyptus urophylla* **(C,D)**, and *Eucalyptus camaldulensis* **(E,F)** seedlings under K partial replacement by Na (0, 0.22, 0.44, 0.67, and 0.90 mmol_*c*_ dm^– 3^ of Na) in well-watered (WW) and water-stressed (WS) conditions.

In *E. saligna* (Figure 6A), the total variance was explained by 64% (PC1 + PC2), with PC1 being explained by *Ψ*w_*N*_, UE_*K*_, [K], and δ^13^C‰, while PC2 was explained by TDM, Std_*AB*_, and [Na]. The parameters *A*, WUE, LA, *E*, and *g*_*s*_ contributed with average weights to explain the data variance in PC1 and PC2. Under WW, low to intermediate Na rates (up to 0.44 mmol_*c*_ dm^–3^) were characterized by higher values of TDM and Std_*AB*_ and lower values of Na and δ^13^C‰. Plants under lower Na rates and WS showed higher WUE and δ^13^C‰ values. The Na rate of 0.9 mmol_*c*_ dm^–3^ resulted in higher Na levels and lower K, TDM, and Std_*AB*_ values.

In *E. urophylla* (Figure 6C), the total variance was explained by 66% (PC1 + PC2), with PC1 being explained by *g*_*s*_ and *E* and PC2 by WUE, δ^13^C‰, [K], TDM, and Na. The parameters Std_*AB*_, UE_*K*_, *A*, *Ψ*w_*N*_, and LA contributed with average weights to explain the data variance in PC1 and PC2. Under WW, low to intermediate Na rates were characterized by higher values of TDM, Std_*AB*_, UE_*K*_, *A*, LA, *g*_*s*_, and *E*. Higher Na rates were identified by higher Na and lower TDM, [K], WUE, and δ^13^C‰ levels. Under WS, Na rates of 0 and 0.22 mmol_*c*_ dm^–3^ resulted in higher WUE and δ^13^C‰ and lower LA, *E*, and *g*_*s*_ levels.

In *E. camaldulensis* (Figure 6E), the total variance was explained by 77% (PC1 + PC2), with PC1 being explained by TDM, *g*_*s*_, LA, *Ψ*w_*N*_, and [Na] and PC2 by WUE, δ^13^C‰, and *E*. The parameters [K], Std_*AB*_, *A*, and UE_*K*_ contributed with average weights to explain the data variance in PC1 and PC2. Under WW, low to intermediate Na rates were characterized by higher values of TDM, *g*_*s*_, LA, *Ψ*w_*N*_, and EU_*K*_ and by lower values of [Na]. Moreover, the Na rate of 0.9 mmol_*c*_ dm^–3^ resulted in higher *E* and [Na] and lower δ^13^C‰, WUE, and [K] values. Plants under WS with low to intermediate Na supply were characterized by higher values of [K], WUE, and δ^13^C‰ and by lower *E* values.

Cluster analysis showed the formation of three main groups among the treatments in all genotypes. In *E. saligna* (Figure 6B), group 1 was represented by Na rates of 0, 0.22, and 0.44 mmol_*c*_ dm^–3^ under WW, which represented the control and low to intermediate rates; group 2 by the rate of 0.67 mmol_*c*_ dm^–3^ under WW and the rates of 0, 0.22, 0.44, and 0.67 mmol_*c*_ dm^–3^ under WS; and group 3 contained K-deficient plants (0.9 mmol_*c*_ dm^–3^ of Na) under both conditions. In *E. urophylla* (Figure 6D), group 1 comprised plants receiving no Na under WW and 0.22, 0.44, and 0.9 mmol_*c*_ dm^–3^ of Na under WS, while group 2 contained plants receiving 0.67 and 0.9 mmol_*c*_ dm^–3^ of Na under WS. In group 3, the plants received 0.22, 0.44, and 0.67 mmol_*c*_ dm^–3^ of Na under WW and no Na under WS. In *E. camaldulensis* (Figure 6F), group 1 was composed of plants receiving 0, 0.22, 0.44, and 0.67 mmol_*c*_ dm^–3^ of Na under WW, while in group 2, plants received 0, 0.22, 0.44, and 0.67 mmol_*c*_ dm^–3^ of Na under WS. In group 3, plants received only 0.9 mmol_*c*_ dm^–3^ of Na under both water regimes.

## Discussion

To withstand drought periods, plants have evolved numerous mechanisms that vary among species (Merchant et al., 2007), which include morphological adaptations such as growth inhibition and stomatal closure (Warren et al., 2007), lowering its LA to avoid overheating (Ahrens et al., 2020) and water loss by leaf transpiration rates (Drake et al., 2019). For this, the plant reduces its tissue water content as a coordination of physiological and structural adaptations (Merchant et al., 2006), as well as cell contraction, turgor loss (Cosgrove, 1997), and slower leaf expansion (Pita-Barbosa et al., 2016). The *Ψ*w_*N*_ inducing stomatal closure plays a critical role in drought avoidance by protecting the integrity of xylem water transport, given that early stomatal closure and leaf shedding precede the beginning of embolism during prolonged drought stress (Li et al., 2020). The drought-induced reductions in plant growth were accompanied by a decrease in LA and leaf gas exchanges, differing in degree among species. Our findings clearly suggest that different genotypes provided adaptations to face drought stress, as indicated by the *g*_*s*_, *Ψ*w_*N*_, and LA reductions, mainly observed in *E. camaldulensis*, showing a higher tolerance to drought stress (Table 1) and confirming *Ψ*w as an effective indicator for measuring the drought tolerance of plants.

Furthermore, plants grown under WW showed the lowest decreases from *Ψ*w_*PD*_ to *Ψ*w_*N*_, demonstrating a great reduction in osmotic pressure to maintain cell turgor in plants grown under WS, which unexpectedly showed higher *Ψ*w_*PD*_ than did plants under WW. Although different hydraulic systems have been found among species and genotypes of the same species (Costa e Silva et al., 2004), a lower *Ψ*w_*PD*_ was expected in WS relative to the WW condition (Drake et al., 2019). The replacement of K by Na in the vacuoles promoted a faster decrease in cell osmotic potential in plants under drought, as it also increased cell expansion in plants under adequate water availability (Hampe and Marschner, 1982). Albeit the three species demonstrated adaptive capacity to the experimental conditions, the values measured differently respond to treatments and water conditions due to the contrasting genotypic patterns that control drought tolerance (Ahrens et al., 2020). The variance in the *Ψ*w values under the WW and WS conditions indicated that the differences in drought tolerance between the *Eucalyptus* species are associated with osmotic adjustments and drought avoidance mechanisms, turning essential the integration of several adaptive strategies simultaneously (Shvaleva et al., 2006). According to the authors, differences in the metabolic responses may also reflect distinct degrees of stress experienced throughout the experimental period. In general, osmolyte accumulation as a consequence of drought reduces the cell osmotic potential and improves the water absorption and cell turgor, sustaining future physiological processes, such as stomatal opening, photosynthesis, cell growth, and enhanced dehydration tolerance under drought conditions. As observed, the drought-avoidance mechanisms of *E. camaldulensis* did not reach the same degree of tolerance to drought stress by *E*. *saligna* and *E*. *urophylla*. Stomata distribution on the leaf surface was also related to the amount of energy used in transpiration (latent heat transfer; Jarvis and McNaughton, 1986). In *E. saligna* and *E. urophylla*, stomata occurred on the underside (hypostomatous leaves), which is common in plants of mesophytic habitats (Figure 1 and Supplementary Figure S1). However, the stomata of *E. camaldulensis*, the drought-tolerant genotype (Gonçalves et al., 2017), occurred on both sides (amphistomatous leaves), which is common in arid environments (Parkhurst, 1978) and has been correlated with a reduced internal diffusion resistance by the lower pathway length of CO_2_ molecules to the carboxylation sites (Mott and Michaelson, 1991). According to the authors, the occurrence of stomata on both sides would be advantageous in plants growing under high light intensity, where the internal CO_2_ concentration limits the photosynthetic rates. As observed in our study, adaptations to drought stress were stimulated in all *Eucalyptus* species with partial K replacement by Na.

An adequate K nutritional status of plants promotes tolerance to abiotic stress (Cakmak, 2005) and enhances the WUE of trees (Battie-Laclau et al., 2016) since water uptake by the roots and stomatal opening are facilitated by K accumulation in the root xylem vessels and guard cells, increasing the tissue’s water status and improving tolerance to water stress (Mengel et al., 2001). According to Ahrens et al. (2020), the WUE is correlated with δ^13^C‰, which in turn relates to leaf gas exchange properties. These statements are in agreement with our findings. Due to drought, the reduction in *E* (biophysical process) as a consequence of the significant decline in *g*_*s*_ was not accompanied in the same degree by *A* (biophysical/biochemical process), increasing WUE, δ^13^C‰, and plant drought resistance (Egilla et al., 2005; Sarabi et al., 2019). In contrast to *E*. *urophylla* and *E*. *camaldulensis*, the drought increased *A* in *E. saligna*, which was unexpected. We hypothesize that evaluations in leaf scale as *A*, *E*, and *g*_*s*_ produce accurate data of a specific time and may not always be reliable in predicting whole plant responses throughout their cycle (Jákli et al., 2016), which in turn can be reflected by δ^13^C‰ and WUE (Condon et al., 2002), integrative indicators of changes in the environmental conditions that occur during the entire experimental period (Seibt et al., 2008). Furthermore, drought stress reduced EU_*K*_ and EU_*Na*_, being an adaptive strategy favoring nutrient accumulation in an unfavorable soil–climate situation to subsequently increase nutrient translocation and use under favorable growth conditions (Müller et al., 2017).

Our studies indicate that, to a certain degree, the replacement of K by Na promoted *Eucalyptus* growth (Subbarao et al., 1999; Krishnasamy et al., 2014), with a small amount of Na being equivalent to K in their function (Ivahupa et al., 2006). Plants with low K replacement by Na showed higher TDM compared to those receiving only K (0 mmol_*c*_ dm^–3^ of Na) or of K-deficient plants (0.9 mmol_*c*_ dm^–3^ of Na) even in those under WS, except for *E. saligna*, the drought-sensitive genotype (Table 1). As Na can partially substitute K in the vacuole, making more K available to the cytosol (Rodríguez-Navarro and Rubio, 2006), low K replacement by Na contributed to enhancing cell turgor and expansion (Wakeel et al., 2011), promoting plant growth (Martínez et al., 2005; Ma et al., 2011; Schulze et al., 2012; Battie-Laclau et al., 2013), as observed by the higher TDM concomitant to the lower *Ψ*w_*N*_ in plants. Furthermore, the estimated ideal percentage of substitution increased according to the drought tolerance of the genotypes, reaching 30% in *E. camaldulensis* under WW, confirming Na as a beneficial element in plant dry matter (Subbarao et al., 2003; Idowu and Aduayi, 2006; Wakeel et al., 2011; Kronzucker et al., 2013) even under drought (Yue et al., 2012; Xi et al., 2018). The leaf K/Na ratio is commonly used as a predictor of plant performance (Munns and Tester, 2008), varying among *Eucalyptus* genotypes (Marcar and Termaat, 1990). An appropriate leaf K/Na ratio was found for low K replacement by Na, as observed by the estimated maximum yield, varying from 1.5 to 3.1 among the *Eucalyptus* genotypes (Marcar and Termaat, 1990) and water regimes (Table 1). A similar leaf K/Na ratio of 3.4 was found by Mateus et al. (2019) in hybrid *Eucalyptus* subjected to K replacement by Na in the nutrient solution. This indicates that Na can reduce the critical levels of leaf K under an adequate K/Na ratio, providing changes in plant performance and demand, albeit without any symptoms of K deficiency (Besford, 1978; Krishnasamy et al., 2014). There is no evidence of key cytosolic components being hampered by a low Na supply, unlike under salinity conditions (Gattward et al., 2012), although a greater efficiency in the osmotic function of plants supplied with both K and Na was observed in our study, corroborating the results of previous studies (Jeschke, 1977). These authors suggested that the replacement of K by Na in the process of osmoregulation in vacuoles improved turgor and cell expansion (Pi et al., 2014). In our study, the low K replacement by Na (up to 50%) increased Std and, consequently, *A, E*, and *g*_*s*_ at the expense of higher WUE values, evidencing the benefits of nutrient interaction to a certain degree.

Low K replacement by Na confirmed that nutrient interaction is a strategy to increase the UE_*K*_ under low soil K availability, proposed by Laclau et al. (2003), not only allowing the maintenance of productivity despite the lower K supply but also favoring plant development (Ma et al., 2011) and allowing the increase in plant TDM. Notably, large proportions of substitution decreased the K/Na ratio and led to lower photosynthetic performance and biomass, as observed by the negative relationship between TDM and Na (Figure 6), evidencing the interaction among mineral nutrition, nutrient use, and soil water availability in *Eucalyptus* (Tariq et al., 2019). Potassium decreased concomitantly to the higher Na rates in *Eucalyptus* (Figure 5), suggesting that the Na ions were directed toward the vacuole as an alternative inorganic osmoticum (Flowers and Lauchli, 1983), including guard cells (Terry and Ulrich, 1973), and releasing K to the cytoplasm and metabolic pathways, such as in the chloroplast (Speer and Kaiser, 1991), stimulating photosynthesis (Krishnasamy et al., 2014) and water retention (Xi et al., 2018). It is widely hypothesized that despite the drop in K, the K cytoplasm concentration is maintained near 100 mmol L^–1^ K, which is required for adequate enzyme activities (Britto and Kronzucker, 2008), as also suggested by Gattward et al. (2012).

However, the stomata of plants under high K replacement by Na cannot function properly, favoring stomatal opening and promoting *E*. The absence of K stimulates ethylene synthesis (Benlloch-Gonzalez et al., 2010), impairing the action of abscisic acid on the stomata, decreasing *g*_*s*_ and delaying stomatal closure (Tanaka et al., 2006), thus reducing WUE and TDM (White et al., 2009; Christina et al., 2018). The LA development was also directly affected by the plant mineral nutritional status (Marschner, 2012), especially K nutrition (Egilla et al., 2005; Battie-Laclau et al., 2013; Tavakol et al., 2018). In our study, yellowing in the leaf margins and even delayed stomatal closure were noticed in K-deficient plants (0.9 mmol_*c*_ dm^–3^ of Na) (Wang et al., 2013), dramatically reducing LA (Bednarz et al., 1998). Plant yield also decreased due to K deficiency, an essential element that cannot be completely replaced (Arnon and Stout, 1939) due to its specific functions, such as enzyme activation, initiation (Spyrides, 1964), elongation (Lubin and Ennis, 1964), termination of translation (Näslund and Hultin, 1971), and conformation of ribosomes (Klein, 2004). Our results indicated that since K-deficient plants occurred at Na rates higher than 0.67 mmol_*c*_ dm^–3^, a K/Na ratio of 1:0 is critical for *Eucalyptus* growth since lower ratios significantly decreased plant TDM. Thus, understanding the K/Na ratio mechanisms may be useful for the development of strategies to reduce K fertilization by replacing it with more cost- and energy-efficient alternatives (Benito et al., 2014).

Drought results in *g*_*s*_ and *g*_*m*_ impairments (Chaves et al., 2009), decreasing *C*_*i*_ and resulting in the fixation of available CO_2_ molecules. Thus, under drought, stomatal closure leads to the enrichment in ^13^C and, consequently, in a higher δ^13^C‰ (Robinson et al., 2000). In contrast, the decrease in δ^13^C‰ indicated higher stomatal aperture (Farquhar et al., 1989), as shown by the lower WUE, confirming that the stomata of K-deficient plants cannot function properly, favoring stomatal opening and promoting *E*. Therefore, K-deficient plants under both conditions were characterized by lower δ^13^C‰, WUE, TDM, and K levels and higher Na levels, which explains their grouping in the same cluster (0.9 mmol_*c*_ dm^–3^ of Na under WW and WS) (Figure 6). Low K replacement, markedly under drought, also resulted in lower numbers of open stomata, indicated by the higher δ^13^C‰ (more positive) and WUE values, which was associated with a better response to drought, confirming the statement that the richer plants are in δ^13^C‰, the greater the WUE, as proposed by Farquhar et al. (1989). Our results also showed greater relative whole plant transpiration than the control plants, suggesting adequate stomatal closure by osmotic adjustment to avoid water loss at the expense of photosynthetic restriction and mitigating drought impacts. Moreover, plants with low replacement levels of K by Na were grouped into the same cluster, with similar responses characterized by higher Std, UE_*K*_, δ^13^C‰, WUE, TDM, and K levels and lower Na levels.

## Conclusion

Regardless of the water condition, the substitution of K by Na at a level of 25–50% reduced the critical level of K without symptoms of K deficiency and allowed optimum *Eucalyptus* dry matter production. It also improved CO_2_ assimilation, Std, UE_*K*_, and WUE and maintained leaf turgidity by reducing *Ψ*_*WN*_, with the stomata partially closed, indicated by the higher δ^13^C‰, mitigating the negative impacts of drought. Furthermore, the estimated ideal percentage of substitution increased according to the drought tolerance of the genotypes (*E. saligna* < *E. urophylla* < *E. camaldulensis*). When only Na was supplied, inferring K-deficient plants, in addition to the lower growth, plants were characterized by lower δ^13^C‰, WUE_*L*_, and K levels and higher Na levels. The ideal leaf K/Na ratio to provide the maximum yield varied from 1.7 to 3.2 among genotypes and water regimes; values below 1:0 were critical for *Eucalyptus* growth since lower ratios significantly decreased plant development.

## Data Availability Statement

All datasets generated for this study are included in the article/Supplementary Material, further inquiries can be directed to the corresponding author/s.

## Author Contributions

All authors contributed to the literature search, discussion, and writing of the manuscript. NM, AVF, JG, and JL conceived and designed the study. NM performed most experiments. ALF and ES assisted with the management of pot culture and plant material, analysis, and interpretation of data. All authors checked and approved the submitted version.

## Conflict of Interest

The authors declare that the research was conducted in the absence of any commercial or financial relationships that could be construed as a potential conflict of interest.

## Footnotes

1 https://imagej.nih.gov/ij/

## Abbreviations

Std_*AD*_: adaxial stomatal density of leaf surface
Std_*AB*_: abaxial stomatal density of leaf surface
*A*: CO_2_ assimilation rate
*g*_*s*_: stomatal conductance
*E*: transpiration rate
δ^13^C ‰: leaf carbon isotopic composition
*Ψ*w: leaf water potential
K: potassium
LA: leaf area
WUE: water use efficiency
UE: use efficiency
WW: well-watered
WS: water stressed.
