# Corrigendum: Partial Substitution of K by Na Alleviates Drought Stress and Increases Water Use Efficiency in *Eucalyptus* Species Seedlings

**DOI:** 10.3389/fpls.2021.689963

**Published:** 2021-05-05

**Authors:** Nikolas de Souza Mateus, Antônio Leite Florentino, Elcio Ferreira Santos, Alexandre de Vicente Ferraz, José Leonardo de Moraes Goncalves, José Lavres

**Affiliations:** ^1^Stable Isotope Laboratory, Center for Nuclear Energy in Agriculture, University of São Paulo, Piracicaba, Brazil; ^2^Applied Ecology Laboratory, Department of Forest Sciences, Luiz de Queiroz College of Agriculture, University of São Paulo, Piracicaba, Brazil; ^3^Federal Institute of Mato Grosso Do Sul, Nova Andradina, Brazil; ^4^Institute of Forest Research and Studies, Piracicaba, Brazil

**Keywords:** sodium application, drought, stable carbon isotope, water use efficiency, water consumption, photosynthesis

An author name was incorrectly spelled as “Antonio Florentino Leite.” The correct spelling is “Antônio Leite Florentino.”

Further, in the original article, we neglected to include the funder “São Paulo Research Foundation (FAPESP),” “2021/00463-7” to “José Lavres.”

The authors apologize for these errors and state that these do not change the scientific conclusions of the article in any way. The original article has been updated.

