# Supplementary material for: Partial Substitution of K by Na Alleviates Drought Stress and Increases Water Use Efficiency in Eucalyptus Species Seedlings
Source: Front Plant Sci. 2021 Mar 15;12:632342. doi: 10.3389/fpls.2021.632342 (PMC8005639; doi:10.3389/fpls.2021.632342)
Supplement: Supplementary file 1 [file Data_Sheet_1.PDF]

Supplementary Material

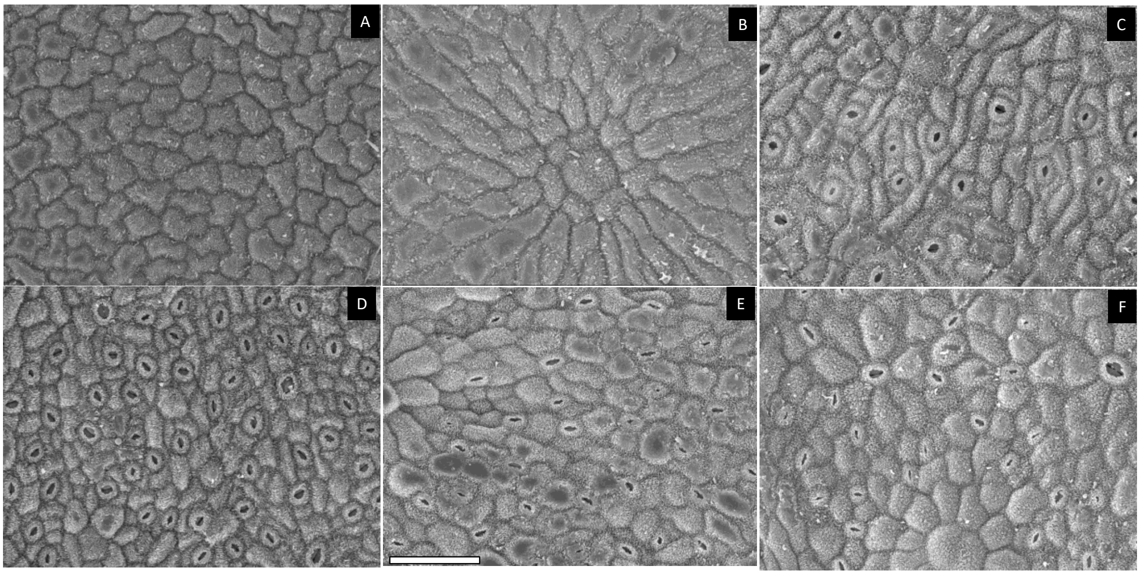

Supplementary Figure 1- Micrograph augmented 500x of scanning electron microscopy in adaxial (A-C) and abaxial (D-F) of *Eucalyptus saligna* (A and D), *Eucalyptus urophylla* (B and E), and *Eucalyptus camaldulensis* (C and F) supplied with 0 $mmol_c$ dm^{-3} of Na under WW condition. Scale bars 50 μM.
